# Epsom Salt-Based Natural Deep Eutectic Solvent as a Drilling Fluid Additive: A Game-Changer for Shale Swelling Inhibition

**DOI:** 10.3390/molecules28155784

**Published:** 2023-07-31

**Authors:** Muhammad Hammad Rasool, Maqsood Ahmad

**Affiliations:** Department of Petroleum Geosciences, Universiti Teknologi Petronas, Seri Iskander 31750, Malaysia

**Keywords:** Deep Eutectic Solvent, NADES, diffused double layer, shale inhibitor, drilling mud

## Abstract

Shale rock swelling poses a significant challenge during drilling a well, leading to issues related to wellbore instability. Water-based mud with specific shale inhibitors is preferred over oil-based drilling mud due to its lower environmental impact. Recently, ionic liquids (ILs) have emerged as potential shale inhibitors due to their adjustable properties and strong electrostatic attraction. However, research has shown that the most commonly used class of ILs (imidazolium) in drilling mud are toxic, non-biodegradable, and expensive. Deep Eutectic Solvents (DESs), the fourth generation of ionic liquids, have been proposed as a cheaper and non-toxic alternative to ILs. However, ammonium salt-based DESs are not truly environmentally friendly. This research explores the utilization of Natural Deep Eutectic Solvent (NADES) based on Epsom salt (a naturally occurring salt) and glycerine as a drilling fluid additive. The drilling mud is prepared according to API 13B-1 standards. Various concentrations of NADES-based mud are tested for yield point, plastic viscosity, and filtration properties for both aged and non-aged samples. The linear swell meter is used to determine the percentage swelling of the NADES-based mud, and the results are compared with the swelling caused by KCl- and EMIM-Cl-based mud. FTIR analysis is conducted to understand the interaction between NADES and clay, while surface tension, d-spacing (XRD), and zeta potential are measured to comprehend the mechanism of swelling inhibition by NADES. The findings reveal that NADES improves the yield point and plastic viscosity of the mud, resulting in a 26% reduction in mudcake thickness and a 30.1% decrease in filtrate volume at a concentration of 1%. NADES achieves a significant 49.14% inhibition of swelling at the optimal concentration of 1%, attributed to its ability to modify surface activity, zeta potential of clay surfaces, and d-spacing of clay layers. Consequently, NADES emerges as a non-toxic, cost-effective, and efficient shale inhibitor that can replace ILs and DESs.

## 1. Introduction

Shale behaves as both a source rock and reservoir rock; i.e., it is able to generate hydrocarbons, and it can offer an environment for trapping these hydrocarbons in a porous medium [1]. Shale contains clay minerals which causes the shale formation to become sensitive towards hydration [2]. There are several types of clay minerals present in the shale formation, e.g., smectite. The smectite is composed of montmorillonite which has tendency to swell in water while kaolinite and illite minerals tend to disperse in water [3,4]. Smectite and smectite/illite mixed layer minerals possess larger specific surface area and high imbibing abilities. The shale containing these two minerals has a strong ability to permeate which will lead to shale swelling causing wellbore instability [5]. Wellbore instability results in non-productive time (NPT), mainly during drilling the shale formations, and results in various problems. These problems are not limited to a stuck pipe, bit balling, lost circulation, caving, and a tight hole, which requires additional time and cost for maintenance [6].

Oil-based drilling fluid (OBDF) is usually preferred to drill a shale-bearing formation because water-based drilling mud (WBDF) will result in shale swelling and ultimately wellbore instability [7,8]. But a higher price and environmental risks are associated with the usage of OBDF which constrains its application. Synthetic-based drilling fluid (SBDF) is also recommended for drilling shale formation, but its ineptness at a higher temperature does not make it an appropriate candidate to be used [9]. WBDF is relatively safe, does not cause grave environmental perils, and has minimal preparation cost which makes it a potential candidate to replace OBDF and incompatible SBDF [10]. Different additives such as shale inhibitors are added into the WBDF to enhance its shale inhibition capability. Shale has a negatively charged clay layer, and these inhibitors bond with that clay layer and neutralize the charge thus stabilizing it against hydration. Traditional inhibitors in water-based drilling fluids include KCl, limes, silicates, polymers, etc., but they are effective to a certain limit [11]. However, apart from a lack of efficiency, a huge concentration of K^+^ (in KCl inhibitor) is not ecologically friendly. Moreover, silicates are highly pH-dependent which also limits their applications [12,13].

Many research groups started exploiting the application of ionic liquids as drilling fluid additives and found out that ionic liquids have successfully improved mud rheology and inhibited shale swelling and hydrate formation [14]. However, recent research has shown that ionic liquid comprising imidazolium-based cations is usually toxic [15,16,17]. Moreover, generally, ionic liquids are expensive, non-biodegradable, and not easy to prepare. This is when the quest to find a cheaper and non-toxic but equally effective alternative to ionic liquids started. This search of researchers concluded with the introduction of Deep Eutectic Solvents (DESs) in many applied fields. A eutectic system is mainly a homogeneous mixture of ingredients that solidifies or fuses at a single temperature that is smaller than the melting point of any of the constituents. This (melting) temperature is regarded as the eutectic temperature. It is the smallest possible melting temperature of all the mixing ratios for the involved constituents.

DESs are simply the mixture of Hydrogen Bond Donor (HBDs) and Hydrogen Bond Acceptor (HBAs) which are mixed at a particular composition (molar ratios) at a particular temperature to obtain a eutectic mixture [18]. Theoretically, it has been generally assumed that the noteworthy decline in melting of a eutectic mixture as compared to that of the pure materials’ is due to the charge delocalization from an HBA to the HBD which is aided by a hydrogen bond (H-bond) formation [19]. Furthermore, the enhanced strength of the hydrogen bonds developed at a eutectic point must be balanced by a reduction in strength of several other cohesive force of interactions. Also, the other crucial factors such as the lattice energies of the HBA and HBD, the changes in entropy upon DES formation, and the way the anion and HBD interact also play a critical role in melting point depression of DES [20]

Ofei et al. (2017) added 1-butyl-3-methylimidazolium chloride (BMIM-Cl) into water-based drilling mud which in turn reduced mudcake thickness up to 50% and decreased YP/PV at all considered temperatures [21]. Han Jia (2019) for the very first time utilized propoanoic acid ChCl (1:1), 3-phenyl propanoic acid ChCl (1:2), and 3-mercapto propanoic acid + Itaconic acid + ChCl (1:1:2)-based DES and achieved **68%**, **58%**, and **58%** bentonite swelling inhibition, respectively [22]. Huang et al. (2020) utilized ionic liquids, i.e., 1-hexyl-3-methylimidazolium bromide and 1,2-bis(3-hexylimidazolium1-yl) ethane bromide, using Na-Bt pellets and achieved **86.43%** and **94.17%** reduction in shale swelling, respectively [23]. Yang et al. (2017) utilized 1- Vinyl-3- ethylimidazolium bromide and achieved **31.62%** reduction in shale swelling with a **40.60%** shale recovery rate [24]. Luo et al. (2017) used 1-octyl-3- methylimidazolium tetraflouroboreate which resulted in an **80%** reduction in shale swelling [25]. M.H. Rasool (2021) utilized Glycerine: Potassium Carbonate DES 2:1 for a free style experiment using swelling shale samples and obtained 87% swelling inhibition [26]. Jingua Ma (2020) used Urea: ChCl and achieved 67% shale swelling inhibition [7].

Though DESs are considered to be the greenest alternative of ionic liquids, in reality, DESs too are composed of some toxic components such as hydrogen bond acceptors, e.g., ammonium-based salts, etc. [27]. This puts a big question mark on the green nature of DES. This is when the natural derivate of Deep Eutectic Solvents called Natural Deep Eutectic Solvents (NADESs), comprising naturally occurring components, came into the picture [28,29]. NADESs, by definition, are also DESs, but their components are naturally occurring substances and salts such as potassium chloride (KCl), calcium chloride (CaCl_2_), Epsom salt (MgSO_4_·7H_2_O), etc. [30]. There are millions of combination of DES and NADES available; thus, this area needs a lot of consideration to exploit as many combinations as possible to check its efficacy and utilization in various applied fields [31,32,33,34]. NADESs are typically composed of combinations of natural compounds such as organic acids, sugars, amino acids, and polyols, which can be sourced from various agricultural and forestry by-products. Unlike traditional solvents that often rely on petroleum-based chemicals, the use of naturally occurring products significantly reduces the production costs associated with NADESs. Furthermore, the abundance and wide availability of these natural compounds contribute to their cost competitiveness. The raw materials for NADESs can often be obtained at lower costs compared to synthetic solvents, which require complex manufacturing processes and specialized chemicals.

Various researchers have successfully prepared novel combination of NADESs/DESs which have been proven to be effective in various applied fields [35]. In 2013, Naser et al. prepared a novel potassium carbonate-based DES and found its thermophysical properties [36]. This newly prepared DES was then later utilized by many research groups in applied fields, e.g., as a hydrate inhibitor, as a drilling fluid additive, for delignification and nano-fibrillation, etc. [18,26,37]. Jordy Kim et al. prepared ascorbic acid-based NADES and determined its antioxidant properties for various applications [38]. Krister et al. concocted a citric acid-based NADES and found out that it can be successfully employed as a potential excipient in collagen-based products [39]. Liu Y. et al. in their detailed review work summarized the applications of NADESs as extraction and chromatographic media [40]. Misan et al. studied the successful applications of NADESs in the agri-food sector [41]. Rasool et al. (2023) tested the successful applications of ascorbic acid-based NADES as a shale swelling inhibitor [42].

This research will utilize Epsom salt as a hydrogen bond acceptor and glycerine as a hydrogen bond donor, selected based upon M.H. screening criteria [43] for selection of DESs for shale inhibition. M.H screening criteria address two factors for the selection of DESs for shale swelling inhibition, (i) the value of total hydrogen bond count and (ii) polarity of functional group. NADESs will be prepared in-house and will then be utilized as a drilling fluid additive to find drilling fluid properties and swelling inhibition traits. FTIR, XRD, and Zeta Potential will be measured to understand the NADES–clay interaction and swelling inhibition mechanism of the clay.

## 2. Results

### 2.1. In-House Preparation

The HBD (Glycerine) and HBA (Epsom salt) have been mixed at various molar ratios at different temperature by stirring at 100 rpm. The final results of experimentation reveal that the eutectic mixture has been obtained at 50 °C when HBA and HBD are mixed with molar ratios of 1:2. The eutectic mixture will only be achieved at eutectic temperature and eutectic mole ratios. If the stirring up is taking place at hypoeutectic and hypereutectic conditions, either the mixture will turn out cloudy, or the precipitation will form.

At 1:2, 50 °C, a transparent and homogenous liquid is obtained with no precipitation, turbidity, and cloudiness, which confirms that this is a eutectic point and the rest of the compositions were either hypoeutectic or hypereutectic solvents. This experimentation also shows that the preparation of an NADES is mainly controlled by three factors: (i) molar ratio, (ii) temperature, and (iii) mixing speed. Among these three factors, it has been observed that molar ratio is the most significant factor while mixing speed is the least controlling factor in the preparation of DESs or NADESs. The detailed preparation and thermophysical and chemical characterization of this Epsom salt-based NADES can be studied in our previous work [44].

### 2.2. Yield Points (YP) and Plastic Viscosity (PV) Ratio (YP/PV)

**Figure 1:**
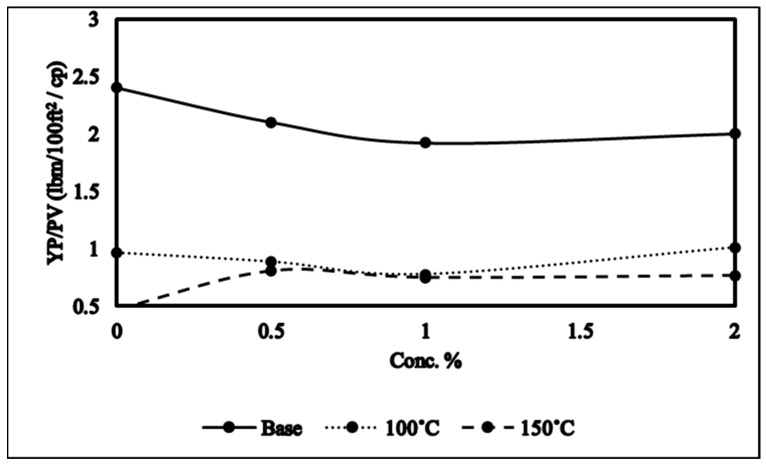
YP/PV of NADES-based mud.

The yield point is described as the force of attraction present between colloidal particles of the drilling mud while plastic viscosity is the resistive force caused by solid particles and the liquid in the drilling mud. The cutting carrying capability of the mud can be best described by YP/PV which is an important indicator of mud rheology. The rise in YP/PV values usually levels the mud flow profile which in turn improves the cutting carrying ability of the mud as shown in Figure 1. It is also worth noting that very high values of YP/PV will result in a surge in annular frictional pressure losses, consequently increasing the equivalent circulation density (ECD), which may result into fracturing. A literature survey has shown that YP/PV values ranging between 0.75 and 1 lbm/100 ft^2^/cp will result in a good cutting transportation without causing any unwarranted ECD. It can be seen from Figure 1 that the addition of NADES in the mud improves the cutting carrying ability of the mud by reducing the YP/PV value nearer to the optimum range. This is because NADESs, just like DESs and ionic liquids, modify the structure of clay platelets which modifies the mud rheology. For aged samples at high temperatures, it can be seen in Figure 1 that YP/PV nearly lies in the optimal range.

The diminution in yield point and plastic viscosity ratio of the drilling mud with the increase in temperature is the combined effect of various phenomena occurring concurrently such as variations in the electrical double-layer thickness surrounding the clay particles, waning in the degree of hydration, augmented thermal energy of the clay particles, and decline in the viscosity of the colloidal medium along with the increase in clay particles’ dispersion. At high temperature, bentonite suffers severe dehydration, mechanical shearing, and degradation. The clay platelets, at high temperatures, after enduring degradation, come closer to each other, and attractive forces are created between them giving the clay platelets a face-to-edge orientation. The increase in temperature will then lead to a state of agglomeration and flocculation that results in lower rheological properties as seen in Figure 1.

### 2.3. Gel Strength

Gel strength helps to assess the mud’s ability to retain suspended cuttings under static conditions. Gel strength is typically measured at three different time intervals: 10 s, 10 min, and 30 min. While both gel strength and yield point are measured in the same units (lb/100 ft^2^), it is important to note that yield point is a dynamic property, whereas gel strength can be considered a static property of the drilling mud. To rationalize results of gel strength, the increase in gel strength at a greater waiting time (10 min) is compared with the previous reading at 10 s. A larger difference between readings indicates a higher pump pressure required to disrupt the gel and initiate circulation. In some cases, the mud may solidify, necessitating the addition of different chemicals to dilute it.

**Figure 2:**
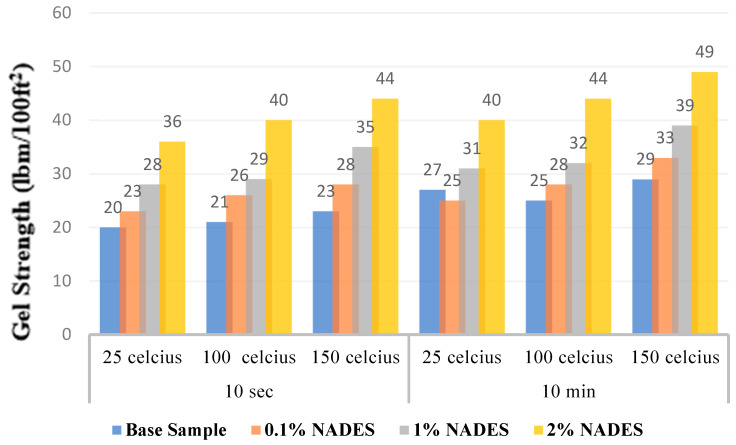
Gel strength profiles of NADES-based aged and non-aged mud samples at 10 s and 10 min intervals.

Analyzing Figure 2, it becomes evident that the incorporation of NADESs into the drilling mud did not lead to a significant increase gel strength. This observation remains consistent for both fresh and aged samples. The presence of NADESs in the samples hindered the increment in gel strength to a notable extent. Notably, the samples containing 1% NADESs exhibited the most substantial decline in the increment of gel strength. It is important to consider these results when evaluating the potential impact of NADESs and DESs on the performance and behavior of the drilling mud. The observation of a good flat profile for gel strength results in the presence of NADESs that can be attributed to several factors. Firstly, NADESs, being composed of naturally occurring compounds, often exhibit good compatibility and interaction with the components of the drilling mud. This compatibility can contribute to the formation of a stable gel network within the mud, leading to a consistent and flat gel strength profile. Additionally, NADESs may act as effective dispersants or stabilizers in the drilling mud system. By reducing the aggregation or flocculation of particles, NADESs can help maintain a uniform distribution of solids, preventing the formation of localized regions with higher or lower gel strength values.

### 2.4. Filtration Properties

**Figure 3:**
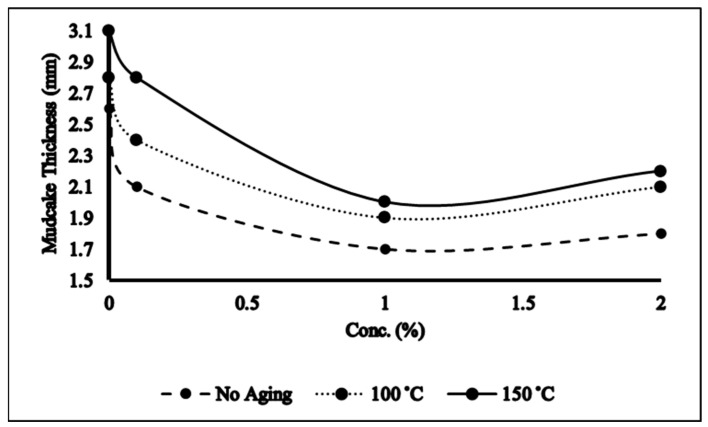
Mudcake thickness of NADES-based mud.

**Figure 4:**
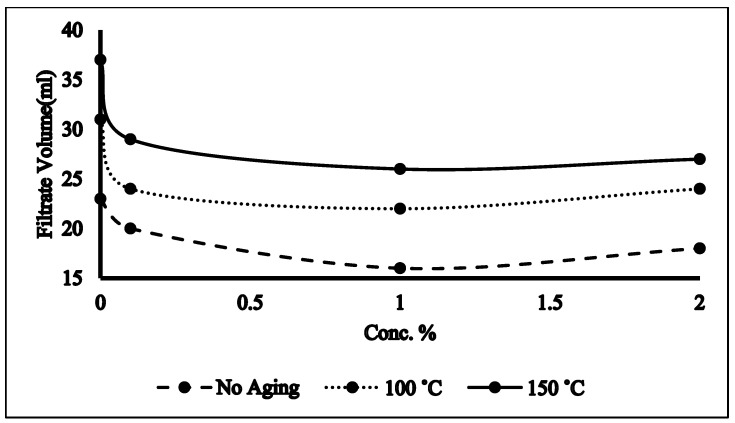
Filtrate volume of NADES-based mud.

Epsom salt–NADES-based mud performed astonishingly under HTHP conditions. As compared to the base sample (no NADES), the addition of NADESs improved the filtration properties of the samples by reducing the mudcake thickness and filtrate volume as evident in Figure 3 and Figure 4. If the mudcake becomes very thick, it will lead to certain problems such as the drill pipe sticking, which causes NPT and wellbore failure. In such scenarios, thinners are added in the drilling mud to reduce the thickness of the mudcake. The NADESs in the mud mimicked the role of a thinner as it decreased the mudcake and filtrate volume. Moreover, this attribute of the NADES may be associated with its ability to bond with clay platelets and change its wettability which in turn will modify the filtration properties of the mud. One percent NADES-based mud with no aging showed the minimum decline of 26% and 30.4% in mudcake thickness and filtrate volume as shown in Figure 3 and Figure 4. The elevated aging temperature caused the mud to flocculate and aggregate which leads to thicker mudcake and higher filtrate loss which is also evident from Figure 3 and Figure 4. It is interesting to note that NADES-based aged mud samples still perform better in terms of filtration properties as compared to the aged base sample while giving optimum results at 1% NADES concentration. The NADES can interact with solid particles present in drilling mud, encapsulating them and aiding in the formation of a filter cake on the wellbore. At lower concentrations, there might not be enough NADESs to effectively encapsulate and hold the solids together, resulting in poor filter cake development and reduced filtration efficiency. Conversely, at higher concentrations, an excessive amount of NADESs might lead to the formation of a dense and impermeable filter cake that hinders fluid flow. The optimum concentration in Figure 3 indicates the range where the NADES concentration promotes optimal solids encapsulation and filter cake formation, facilitating efficient filtration.

### 2.5. Shale Swelling Inhibition

**Figure 5:**
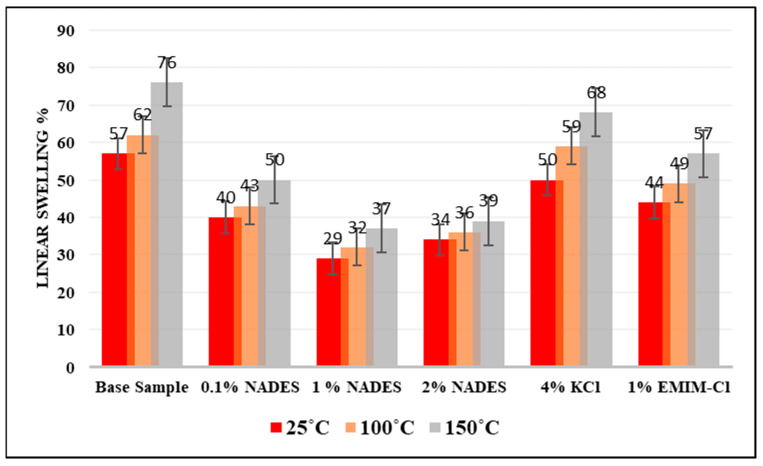
Comparison of Shale swelling caused by NADES, KCl, and EMIMCl.

The percentage increase in swelling has been found for all inhibitor-based samples and compared with the base drilling fluid sample. Figure 5 shows that the base sample without any inhibitor resulted in a 57% increase in shale swelling. The swelling decreased to 50% when KCl is added into the mud sample which further decreased to 44% for EMIM-Cl. The swelling for NADESs at 1% further dropped to 29%.

All inhibitors are able to inhibit shale swelling to some extent. KCl inhibited shale swelling up to 12%; EMIM-Cl inhibited shale swelling up to 22.8%; and NADES inhibited shale swelling up to 49.12% as shown in Figure 4. KCl mainly inhibits shale swelling by the cationic exchange between clay layers. It expels the water cation between the clay layers and makes the clay relatively stable. KCl-based drilling mud has shown the lowest swelling inhibition property. This is because KCl needs to be used in a very high concentration to give optimal results, which outweighs its advantages. Ionic liquids possess a strong electrostatic force of attraction and are able to interact with clay with an electrostatic force of attraction, thus neutralizing the clay, which stabilized the shale hydration process. Moreover, the hydrophobic alkyl chain also plays a vital role in the swelling inhibition properties of ionic liquids. Swelling inhibition due to EMIM-Cl is because of its ability to induce hydrophobicity in the medium and maks expulsion water between the alumino-silicate layers of clay easier. However, they are very expensive, and imidazolium-based ionic liquids are toxic and non-biodegradable; therefore, they are not a feasible solution for shale swelling problems.

Conclusively, NADESs gave better results as compared to the other inhibitors, a result which is associated with its excellent hydrogen bond formation capability. NADES interacts with negatively charged clay and attaches onto it, thus neutralizing clay charges, which stabilizes the shale hydration process. Moreover, NADES also replaces water between the clay layers, which is confirmed from the results of d-spacing.

### 2.6. Underlying Mechanism

#### 2.6.1. FTIR of NADES-Based Mud

**Figure 6:**
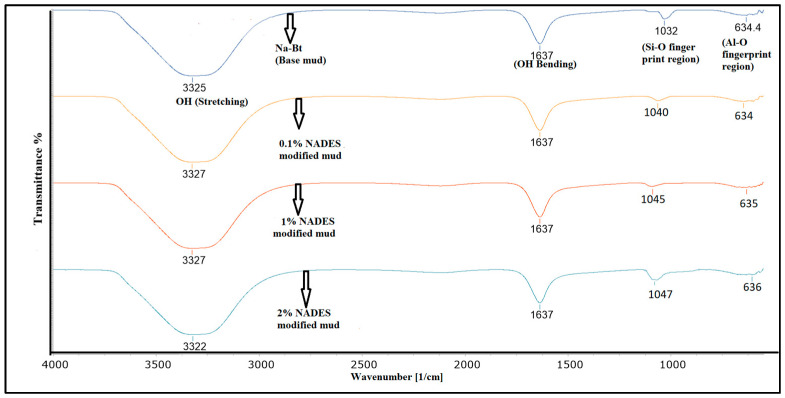
FTIR of NADES-based drilling mud.

FTIR has been conducted to examine and confirm the interaction of clay with different concentrations of NADES. Sodium bentonite (Na-Bt) contains alumina and silica incorporated in its layered caged structure. A measurement of 1032 cm^−1^ is the fingerprint region for alumina while 634 cm^−1^ is the fingerprint region for alumina mineral as shown in Figure 6. NADES has the strong ability to formulate hydrogen bond and the interaction by NADES and Na-Bt clay is shown by change in wavenumbers of silica region for different concentrations of NADES. The change in wavelength corresponds to the change in electronegativity difference which in turn depicts the formulation of new bonds. In the case of NADES, this new electrostatic interaction is hydrogen bonding. Thus, the FTIR confirms that NADES has successfully interacted with clay which in turn explains the modification of rheological and filtration properties of the drilling mud.

#### 2.6.2. Surface Tension

**Figure 7:**
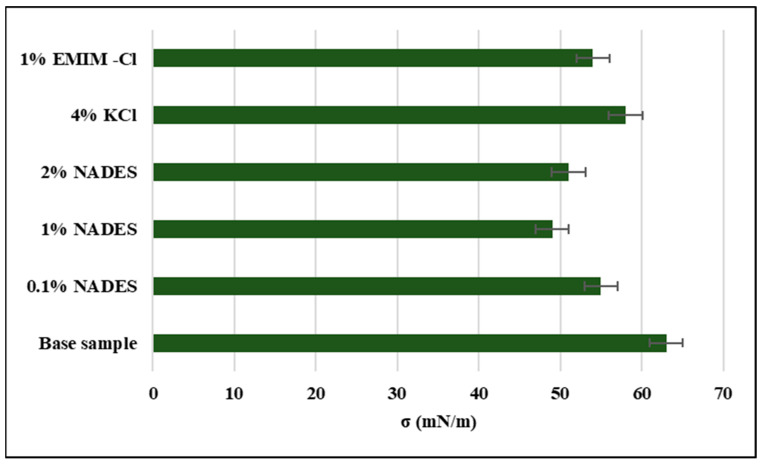
Surface tension of inhibitor-based samples.

The anti-swelling traits of the clay can be well realized by studying the surface tension of the base mud and inhibitor-based mud at various concentrations. Surface tension has a direct proportion to the capillary pressure, though the relation is not very straightforward [45]. The more the capillary pressure will be at the surface of a shale, the higher the tendency will be for water cations to permeate into the clay layers and result in swelling [46]. The shale inhibitors have the tendency to impact surface activity and lower the surface tension, which obstructs the water to permeate into the clay layers. The maximum decline of 23.8% in surface tension had been noted in the case of 1% NADES inhibitor while 14.2%, 19.02%, 6%, and 14.9% declines have been observed for 0.1% NADES, 2% NADES, 4% KCl, and 1% EMIM-Cl, respectively, as shown in Figure 7. The decline in surface tension by NADES is attributed to its strong ability to formulate the hydrogen bond with clay, thus altering the contact angle and capillary behavior which in turn modifies clay behavior in presence of water. The results are in accordance with the results of LSM.

#### 2.6.3. Zeta Potential

**Figure 8:**
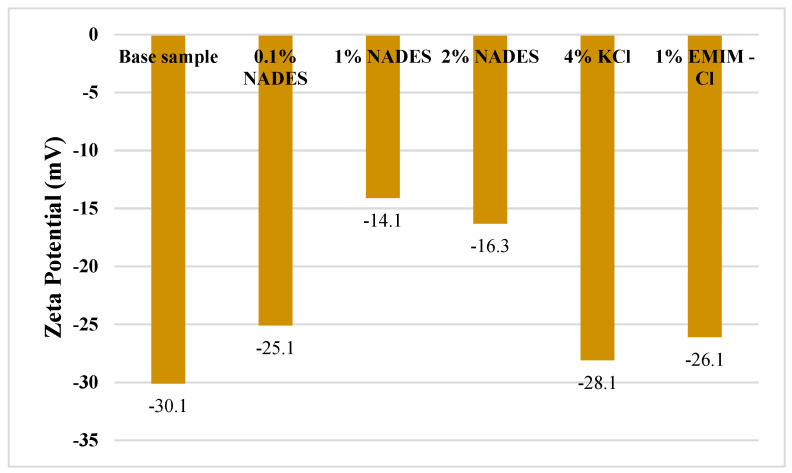
Zeta potential of mud samples.

Zeta potential refers to electrokinetic potential that further corresponds to a dispersion. Clay has a negatively charged surface which is responsible for water cations in clay layers, thus causing swelling. The results of Zeta potential depict the effect of inhibitors on the thickness of an electric double layer of the clay. The figure shows that the inhibitors lessened the electrical double layer which affects the cationic exchange between the inhibitors and the clay. The decline in ZP of 0.1% NADES, 2% NADES, 4% KCl, and 1% EMIM-Cl turn out to be 16.2%, 45.8%, 6.6%, and 13.2%, respectively. The most significant reduction of 53.1% can be observed in the case of 1% NADES which causes the maximum decline in ZP, thus affecting the electrical double layer accordingly as shown in Figure 8. The results of Zeta potential are further supported by the d-spacing results in the next section.

#### 2.6.4. d-Spacing (XRD)

**Figure 9:**
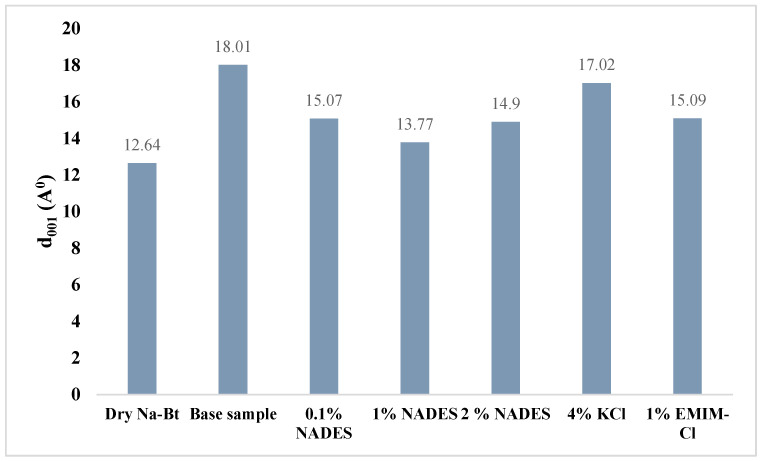
Zeta potential of NADES-, KCl-, and EMIM-Cl-based mud.

d-spacing is the sum of interlayer plus one of the alumino-silicate layer (in clay). The d-spacing of the hydrated samples decreases by the addition of an inhibitor into the base sample, which confirms the intercalation of inhibitors’ cations into the clay layers. The d-spacing of dry Na-Bt comes out to be to be 12.64 A° which increases to 18.01 A° after hydrating the sodium bentonite. After the addition of KCl, the cationic exchange between Na^+^, K^+^, and water cations started which reduces the d-spacing to 16.01 A° as shown in Figure 9.

The 1% NADES inhibitor resulted into the maximum exclusion of water between the clay layers as shown by d-spacing of 13.77 A^o^ while 0.1% NADES, 2% NADES, 4% KCl, and 1% EMIM-Cl result in d-spacing of 15.07 A°, 14.9 A°, 17.02 A°, and 15.09 A°, respectively. This decline in d-spacing values shows that the inhibitors have effectively intercalated between the clay layers and have expelled the water out from the clay layers thus making clay more stable against hydration. This also shows that all added inhibitors have more affinity towards clay than water which makes them apt for shale stabilization, and as per the comparison, 1% NADES has shown the most affinity towards clay as compared to water and proved to be the best inhibitor with optimized concentration in this study. The results of d-spacing are in accordance with results of the linear swell meter.

## 3. Materials and Methods

### 3.1. In-House Preparation of Epsom Salt-Based NADES

Epsom salt and Glycerine have been mixed up at different molar ratios to obtain a eutectic mixture. A eutectic mixture is visually validated by a homogenous, transparent liquid with no turbidness which implies that HBD and HBA have been mixed at eutectic composition. Initial experiments have been conducted to observe the temperature dependance of mixing pattern of HBD and HBA. The eutectic mixture ratios have been assessed at three temperatures 50 °C, 70 °C, and above 100 °C because, as per the literature, for most DESs, eutectic temperature lies between 50 and 80 °C. A METTLER Digital Balance has been utilized to weigh the HBD and HBA while a Thermo Fisher Hot plate has been used for controlled heating and stirring of HBD and HBA at 100 rpm.

### 3.2. Drilling Fluid Composition

The water-based drilling mud has been prepared by using API 13B-1 standards using the composition mentioned in Table 1. Epsom Salt and Gylcerine (99USP) have been procured from Sigma Aldrich, Malaysia, for preparation of NADES. KCl is used as a conventional shale inhibitor and has been obtained from Sigma Aldrich, Subang Jaya, Malaysia. 1-ethyl, 3-methyl imidazolium chloride (EMIM-Cl) > 98% is chosen because it has been found to give excellent results as drilling fluid additives for modifying mud rheology and shale inhibition [47]. KCl and EMIMC-Cl will be used for comparison purposes for shale inhibition traits of NADES.

### 3.3. Bentonite Wafers Preparation for Linear Swelling Test

Various researchers prefer bentonite wafers for shale swelling studies as bentonite contains the same ‘smectite’ group which is responsible for swelling in shale. Moreover, it is not easy to find shale ‘true’ core samples because coring renders shale too unstable and the core samples that are obtained are not 100% shale (may have sandstone and limestone layers mixed). Moreover, swelling inhibition experiments cannot be carried out on shale outcrop because usually they do not contain the smectite group which is responsible for shale swelling.

This research utilizes refurbished pellets of bentonite around 2.54 cm in diameter which are created by compressing a mass of 11.5 g of Na-bentonite powder at 1600 psi using a hydraulic press. The thickness of the pellets is measured before introducing the pallet into LSM environment. These pellets are then dipped in the drilling mud sample (base sample and sample with the inhibitor-based mud), and the difference in the thickness of pellet is later determined by the linear swell meter at the frequency of 60 s for 24 h.

### 3.4. Rheological and Filtration Properties

Different concentrations of NADES-based mud samples (0.1%, 1%, and 2%) have been subjected to FANN Viscometer to note the readings at 3 rpm, 6 rpm, 300 rpm, and 600 rpm after and before aging at 100 °C and 150 °C. For aging, mud samples have been kept in a rolling oven for 24 h at 1000 psia. The resultant values were then used to find the yield point (YP) and plastic viscosity (PV) using Equations (1) and (2). Gel strength of mud samples has been found at 10 s and 10 min intervals.

***Plastic Viscosity (PV)**** = Reading at* 600 rpm − *Reading at* 300 rpm (1)

***Yield Point (YP)**** = Reading at* 300 rpm − *Plastic Viscosity (PV)* (2)

### 3.5. Linear Swell Meter (LSM)

Grace HPHT Linear Swell Meter (M4600) is a sophisticated piece of equipment which can directly report the swelling by measuring the change in thickness of the sample. This study uses an LSM for the evaluation of shale swelling inhibition traits of prepared water-based drilling fluid. This apparatus has two distinct components, i.e., a Wafer Compactor and Linear Swell Meter (Model: M4600). (Bentonite) wafers have been prepared using a Grace core/wafer compactor while swelling tests have been conducted using the main equipment (LSM) which gives the real time swelling data. In this study, a bentonite sample (wafer) has been used for the swelling tests. Procuring shale cores can be challenging, and shale outcrops often lack the specific smectite or S/I (smectite/illite) mixed layer characteristics required for accurate swelling tests. Therefore, utilizing a bentonite sample, which is a type of clay with similar properties to shale’s clay content, allowed us to assess the swelling behavior in a representative manner.

### 3.6. Surface Tension

Surface tension is the tension existing on the liquid surface due to the cohesive forces. The invasion of the water cations into the shale’s micropores is caused by the capillary action which is deemed to be directly proportion to the surface tension. An Optical Contact Angle Measuring Device OCAH 200 has been employed to find the surface tension of the drilling fluid samples. Firstly, the device is set up and calibrated. The sample is prepared and positioned on the stage, ensuring a clean and level surface. The lighting is adjusted, and an image of the sample is captured using the OCAH 200. The image is analyzed using the provided software, with measurement points selected and contact angles measured. The surface tension is calculated using the built-in algorithms of the software, and the process is repeated for multiple measurements, with the results averaged. The data are recorded and analyzed, and the equipment is cleaned after completion. The device’s user manual should be referred to for specific instructions tailored to the OCAH 200.

### 3.7. d-Spacing

d-spacing is the sum of interlayer spacing between alumino-silicate layers in clay and 1 alumino-silicate layer. The wet drilling mud samples with incorporated 0.1%, 1%, and 2% NADES along with 4% KCl and 1% EMIM-Cl have been used to examine the intercalation of all inhibitors into the bentonite layers by using X-ray diffraction (XRD) analysis. A benchtop X-ray diffractometer (D2 phaser) operating at the current of 40 mA and 45 kV with Cu-Kα radiation (λ = 1.54059 Å) has been utilized to obtain XRD peaks for all wet samples along with dry Na-Bt. Bragg’s equation was then applied to find the d-spacing.

### 3.8. Zeta Potential

Malvern Zetasizer Nano ZSP has been to measure Z.P of the diluted drilling mud samples. The sample is prepared and loaded into the cuvette, taking care to remove any air bubbles or contaminants. The instrument settings are configured, and the measurement process is initiated to analyze particle movement in an applied electric field. The zeta potential values and associated data are provided by the Zetasizer software 7.11 for analysis. Multiple measurements are repeated to calculate an average zeta potential value and evaluate measurement variability. The data are interpreted in the context of the specific study or application. Finally, the cuvette or sample cell is cleaned to maintain instrument integrity. The manufacturer’s instructions and user manual should be followed for accurate zeta potential measurements using the Malvern Zetasizer.

### 3.9. FTIR

Fourier-transform infrared spectra (FTIR, Perkin Elmer) of NADES-WBDF (prepared under API 13B/1 standards) have been conducted to analyze the interaction of NADES with the bentonite clay. The prepared samples (NADES modified mud wafers) were loaded onto the sample holder and pressed into pellets for analysis. The FTIR instrument, such as the Perkin Elmer FTIR spectrometer, was utilized with appropriate settings, including a suitable wavelength range and resolution. Each sample was scanned multiple times to obtain an average spectrum and reduce measurement variability. Background subtraction was performed using a reference material to eliminate interference from atmospheric gases. The resulting FTIR curves were then analyzed to identify specific functional groups and chemical components present in the drilling mud samples. Standardized protocols and manufacturer guidelines were followed throughout the process to ensure accurate and reliable FTIR measurements.

## 4. Conclusions

- Different inhibitors inhibit the shale formation by neutralizing the charge on the clay surface, thus making it more stable against hydration.
- NADES-based mud improved YP/PV and filtration properties of the mud which is mainly because of hydrogen bonding between the clay and NADES that modifies the clay structure and thus rheological properties.
- Among KCl, NADES, and ILs, NADES gave the best performance as a shale inhibitor which is attributed to its ability to form hydrogen bonds with clay and thus neutralizing the charge on the clay surface which will hinder the permeation of water cations into the clay layers.
- FTIR shows that NADES has successfully bonded with the silica present in clay. NADES has also decreased surface tension on clay surface which in turn reduced the capillary action and thus reduced the permeation of water cations into clay layers. Moreover, the decline in d-spacing of NADES-based mud shows that NADES possesses more affinity than water toward clay which makes it a potential shale inhibitor against hydration.

## Figures and Tables

**Table 1:** Drilling mud composition.

Component	Weight/Conc/Vol
Na-Bentonite	22.5 g
Soda Carobonate	0.25 g
NaOH	0.25 g
Water	350 mL
NADES (Epsom salt: Gly)	0.1%, 1%, 2% Volume of water
KCl	4% volume of water
1-ethyl-3-methylimidazolium Chloride	1% volume of water

## Data Availability

No data are provided along with this research work.
